# Plasma-Conditioned Liquids as Anticancer Therapies In Vivo: Current State and Future Directions

**DOI:** 10.3390/cancers13030452

**Published:** 2021-01-25

**Authors:** Xavi Solé-Martí, Albert Espona-Noguera, Maria-Pau Ginebra, Cristina Canal

**Affiliations:** 1 Biomaterials, Biomechanics and Tissue Engineering Group, Department Materials Science and Engineering, Escola d’Enginyeria Barcelona Est (EEBE), Universitat Politècnica de Catalunya (UPC), 08930 Barcelona, Spain; xavier.sole.marti@upc.edu (X.S.-M.); albert.espona@upc.edu (A.E.-N.); maria.pau.ginebra@upc.edu (M.-P.G.); 2 Barcelona Research Center in Multiscale Science and Engineering, Universitat Politècnica de Catalunya, 08930 Barcelona, Spain; 3 Research Centre for Biomedical Engineering (CREB), Universitat Politècnica de Catalunya, 08034 Barcelona, Spain; 4 Institute for Bioengineering of Catalonia (IBEC), Barcelona Institute of Science and Technology (BIST), 08034 Barcelona, Spain

**Keywords:** plasma-conditioned liquids, cancer, in vivo, cold atmospheric plasma

## Abstract

**Simple Summary:**

During the last decade, cold atmospheric plasmas (CAP) have been broadly investigated for their therapeutic effect against cancer. CAP sources can be used to treat liquid media, thereby generating plasma-conditioned liquids (PCL). PCL represent a very interesting alternative to direct CAP treatment, because they may allow treatment of malignant tumors located in inner organs of the body by means of an injection, thus avoiding multiple surgeries. Although research on this therapy is still in its early stage, PCL have already demonstrated their potential anticancer effect in different types of cancer in vivo. This review gathers the existing literature involving PCL treatments in vivo, highlighting the differences between the approaches undertaken and the need for establishing standardized protocols in order to better understand the effects of PCL-based therapies in vivo.

**Abstract:**

Plasma-conditioned liquids (PCL) are gaining increasing attention in the medical field, especially in oncology, and translation to the clinics is advancing on a good path. This emerging technology involving cold plasmas has great potential as a therapeutic approach in cancer diseases, as PCL have been shown to selectively kill cancer cells by triggering apoptotic mechanisms without damaging healthy cells. In this context, PCL can be injected near the tumor or intratumorally, thereby allowing the treatment of malignant tumors located in internal organs that are not accessible for direct cold atmospheric plasma (CAP) treatment. Therefore, PCL constitutes a very interesting and minimally invasive alternative to direct CAP treatment in cancer therapy, avoiding surgeries and allowing multiple local administrations. As the field advances, it is progressively moving to the evaluation of the therapeutic effects of PCL in in vivo scenarios. Exciting developments are pushing forward the clinical translation of this novel therapy. However, there is still room for research, as the quantification and identification of reactive oxygen and nitrogen species (RONS) in in vivo conditions is not yet clarified, dosage regimens are highly variable among studies, and other more relevant in vivo models could be used. In this context, this work aims to present a critical review of the state of the field of PCL as anticancer agents applied in in vivo studies.

## 1. Introduction

Plasma-treated or plasma-conditioned liquids (PCL), often designated also as plasma activated liquids in the literature, are produced when the reactive oxygen and nitrogen species (RONS) generated from a cold atmospheric plasma (CAP) source are transferred to a liquid. The transfer of these reactive species to the liquid is the result of complex reactions between the plasma and the liquid at the plasma–liquid interface [1,2]. PCL can contain superoxide anions (O_2_^−^), hydroperoxyl radicals (HOO·), hydrogen peroxide (H_2_O_2_), hydroxyl radicals (·OH), atomic oxygen (O), singlet oxygen (^1^O_2_), ozone (O_3_), nitric oxide (NO), nitrogen dioxide (NO_2_), peroxynitrite (ONOO^−^), nitrite (NO_2_^−^), and nitrates (NO_3_^−^), as well as dichloride radicals (Cl_2_^−^·) and hypochlorite anions (ClO^−^) [3,4,5]. Depending on their lifetime, RONS can be classified as short-lived or long-lived reactive species. It is known that short-lived reactive species (i.e., radicals, atomic oxygen, peroxynitrite, etc.) have low stability and a short lifespan (sometimes around a few nanoseconds) and therefore, their chance to reach the targeted cells or tissue is limited [6]. Consequently, due to their higher stability, long-lived and far-ranging reactive species (mainly hydrogen peroxide, nitrites, nitrates, and organic peroxides) are considered to be the major biologically relevant elements in PCL treatments [7].

Research in this field suggests that CAP-generated RONS can be used to target malignant tumors, inducing apoptosis to cancer cells, without damaging healthy cells or tissues [8,9]. In fact, cells produce RONS themselves through intrinsic biochemical processes and, such RONS play a central role in reductive-oxidative biology (redox biology), which is fundamental in the biochemistry of cells [10]. The supply of exogenous RONS has been demonstrated to trigger specific biological pathways in cells [11]. For PCL, this anti-cancer effect is thought to be related to the concentration and type of RONS generated in the liquid upon CAP treatment and, in the last years, very interesting results have been published postulating PCL as a novel and promising therapy for cancer treatment [12].

So far, PCL’s therapeutic effects have been proved against various types of cancer in vitro [13,14] and ex vivo [15], and some researchers have already reported their beneficial effects in vivo [16,17,18,19,20,21,22,23,24,25,26,27,28,29,30,31,32,33] in murine models. However, comparison among the different works dealing with the biological action of PCL in vivo is hard due to the absence of standard protocols. In this context, this review aims to recapitulate the existing literature, from the earlier studies in 2013 [23] until the present date, on the use of PCL in animal models, and to provide a critical review of the methodologies used to treat tumors in vivo, thus shedding light on the next steps forward in the field.

## 2. In Vivo Cancer Model: Animal Model Selection

The use of adequate preclinical in vivo models is one of the most important components in every aspect of translational cancer investigation, ranging from the understanding of the biological basis of the disease to the development of new treatments [34]. Currently, the anticancer activity of PCLs in vivo has only been assessed in mice, which is the most commonly used preclinical in vivo platform in cancer research [35]. There are different kinds of cancer mice models, basic models that rely on grafted tumors derived from cell lines or explants, and more advanced ones with genetically modified animals that are engineered to intrinsically develop the disease on their own [36].

**Figure 1:**
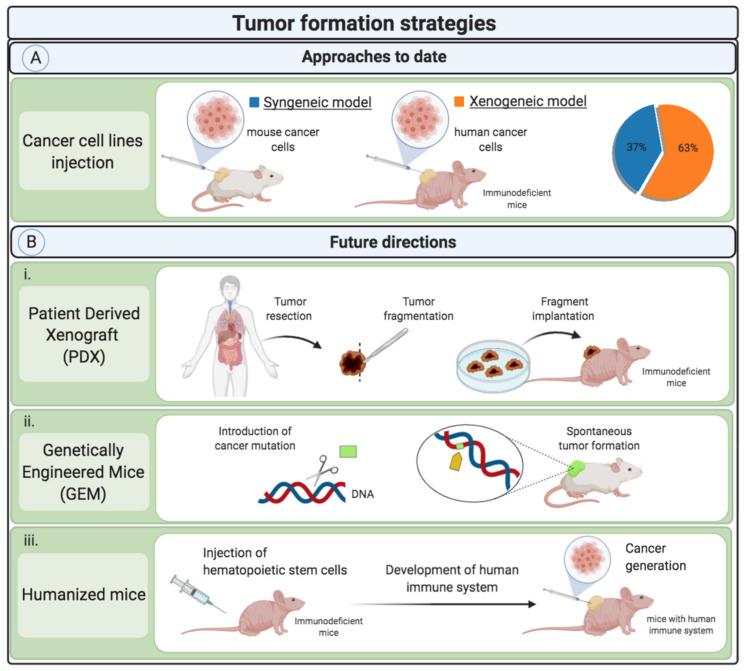
Tumor formation strategies on mice models for PCL treatments in vivo. (**A**) Current approaches assessed in the formation of tumors in animals for PCL treatments in vivo. (**B**) Possible future directions in the generation of tumors in mice for the study of PCL treatments in vivo, which involve (**B.i**) patient derived xenografts, (**B.ii**) genetically engineered mice models, and (**B.iii**) humanized mice models.

Only grafted tumors derived from cell lines have been used to date to investigate the antitumoral potential of PCLs. In 63% of the studies, a xenograft tumor was formed using human cancer cell lines, while in the other 37%, mouse cancer cells were used to generate syngeneic tumors (Figure 1A). Cancer modeling with cell line-derived xeno- and allografted tumors is widely used in preclinical drug screening due to its ease of use, high reproducibility, and relatively low associated costs [37]. Using cell line-based tumors can be a first step for the investigation of the anticancer effect of PCL, but it involves some limitations; for instance, these cells are immortalized and maintained in culture for many generations and, as a consequence, they may lose and/or gain genetic features that can alter their phenotype, biological functions like cell growth and migration, as well as their responsiveness to stimuli [38]. Furthermore, using allogeneic or xenogeneic cell lines, it is not possible to represent the complex molecular variations between patients shown in the clinic that involve differences in malignant growth, invasive and metastatic capacity, and the acquisition of drug resistance [39,40].

In this line, Xiang et al. studied the effect of PCLs in different breast cancer subtypes with two of the most used human breast cell lines, the MDAMB231 and the MCF-7 cells [21]. The MCF-7 cells were isolated from a 69-year-old woman with metastatic breast cancer, while the MDAMB231 cell line was obtained from a 51-year-old patient with the same cancer affliction. Plasma-treated DMEM medium was used as PCL and 200 µL were daily administered for 30 days. Results demonstrated the huge interpatient variability, since MDAMB231-derived tumors responded well to the PCL therapy with a tumor growth reduction of around 80%, while in MCF-7-derived tumors, PCL treatment barely achieved a tumor growth reduction of 10%. This highlights that although the use of cell lines is a valid approach in the first stages of the research for initial screenings, their poor predictive potential may make difficult the translation of PCL-based antitumoral therapies, from more advanced stages of the investigation towards clinical application [41]. To better represent the interpatient tumor heterogeneity and improve the predictive potential of the approach, one possibility in future experiments could be to evaluate the anticancer activity of PCL in patient-derived xenografted tumors (PDX) (Figure 1B.i). These cancer models rely on generating tumors in animals through the implantation of cells or cancer tissues that are directly extracted from the patient’s tumors [42]. It has been shown that PDX are more representative of human cancer biology and that they display a higher correlation with the patient’s specific response to treatments [43,44,45]. In this sense, the PDX platform could allow obtaining valuable and useful information, while enabling personalized medicine with PCL dose regimens adjusted to the patient’s tumor characteristics. However, xenografted tumor models are based on the implantation of cancer cells into immunodeficient mice. It is widely accepted that the immune system is closely related to cancer prognosis, oncogenesis, and response to anticancer therapies. In this sense, the absence of the immune components does not allow studying the implications and effects of PCL over the immune system in cancer models. Therefore, using these models may compromise their predictive value in translational research of PCL in cancer therapy. 

Alternatively, advanced cancer models based on genetically modified animals that develop cancer themselves could also overcome the drawbacks of cell line-derived cancer models [46] (Figure 1(Bii)). These advanced models mimic the physiological and biological tumor environment (extracellular matrix composition, tumor architecture, degree of vascularization, etc.) with higher accuracy compared to the mentioned in vivo cancer models [47] while preserving the immune system of the mice. However, the mice immune system differs from that of humans. In order to study the human immune system in mice models, the so-called humanized mice models have been developed (Figure 1(Biii)). In these models, immunodeficient mice are engrafted with human hematopoietic stem cells that develop into functional human immune systems. Consequently, humanized mice could be used to better investigate the efficacy of PCLs in cancer immunotherapy [48,49,50].

The mentioned approaches (PDX, genetically engineered and humanized animals) require more technical and economic resources, but the use of these advanced cancer models might offer more valuable and translational information from the experimental research.

## 3. PCL Generation for In Vivo Cancer Treatment. Experimental Setup

The outcomes of the CAP-treatment in terms of the RONS generated in a liquid can greatly vary depending on parameters of the experimental setup (device, applied voltage, nature of the gas, gas flow, distance of treatment, composition of the liquid, liquid volume, etc.) [2,51]. Analysis of the literature regarding PCL employed in in vivo studies for cancer treatment puts forward that many differences in the experimental setup have been used to produce and characterize PCL for cancer treatment applications (Table 1).

### 3.1. Relevant Parameters in Plasma Treatment

As reflected in Table 1, plasma jets are the main type of CAP-device employed to produce PCL for biomedical applications, followed far behind by dielectric barrier discharges (DBD) [51]. For in vivo experiments, the working gas most often employed was argon, although some studies used helium or mixtures of helium and oxygen, with gas flows ranging from 0.2–10 slm. However, the most recurrent configurations are set to 2–5 slm. A very similar thing happens with the distance of treatment, whose values range from 2 to 30 mm (distance between the nozzle of the jet and the surface of the liquid). Logically, the smallest distances of treatment are set when using DBD to produce PCL. Nonetheless, distances down to 3 mm are also found in certain plasma jet setups [28,30,31,32]. In all cases, the treatment time of the liquid to generate PCL is in the order of minutes, 1 and 60 min being the shortest and the longest treatment time. These conditions are relevant in how they affect the concentration and type of RONS generated in the PCL.

### 3.2. Reactive Species Generated in PCL

Among the different reactive species generated in the liquid after CAP-treatment, it is believed that the synergistic effect of long-lived RONS such as of H_2_O_2_ and NO_2_^-^ are great contributors to the anticancer therapeutic effects of PCLs [4,52]. Although H_2_O_2_ and NO_2_^-^ are not the only RONS in charge of the lethal effect of PCL on cancer cells [4,6], it is essential to characterize and quantify as thoroughly as possible the RONS in PCL in order to have a better understanding of their biological effects in vivo. For this reason, besides the description of the conditions and parameters used to generate PCL (described in the previous section), a meticulous quantification of RONS should be included, since the lethal concentration of RONS may be different depending on the type and site of the tumor treated.

While certain RONS are hard to detect due to their short lifespan, others, like H_2_O_2_ and NO_2_^−^ or NO_3_^−^, are more easy to detect and quantify with methods that do not require very sophisticated equipment [51,53]. However, only ~35% of the in vitro studies performed prior to the in vivo investigations herein reviewed reported the characterization of RONS in PCL, detecting species such as H_2_O_2_, NO_2_^−^, NO_3_^−^, O_2_, O_3_, or O_2_^−^, depending on the specific work. H_2_O_2_ was the only chemical that is quantified in all cases [19,21,24,25,30,32,33] with concentrations ranging from ~0.01 to ~200 μM in the PCL, and the resulting cocktail of RONS showed effectiveness in killing different types of cancer cells in vitro. However, when moving from in vitro to in vivo studies, we observe disparities between the characterization of RONS that may compromise and difficult the understanding of the biological effects of PCLs.

On the one hand, although many of the works reported accurate concentration of RONS in the PCL used in vitro, longer CAP treatment times were employed to generate PCL for the in vivo condition, and the precise concentrations in the in vivo setting were not reported [21,23,24]. For instance, Tanaka et al. analyzed different saline solutions based on Ringer’s lactate composition (combining NaCl, KCl, CaCl_2_, and L-sodium lactate) reporting differences in the concentration of H_2_O_2_ generated upon CAP treatment. Even though in some liquids, the concentration of H_2_O_2_ was similar, these PCLs showed unequal cytotoxicity on brain tumor cells (U251SP) in vitro, attributing the therapeutic effect on the generation of acetyl and pyruvic acid-like groups due to the presence of L-sodium lactate in the Ringer’s lactate solution. However, as discussed above, the treatment time was longer and the volume of liquid treated was lower for the in vivo assay, thus possibly generating much higher concentrations of RONS. Additionally, it has been observed that smaller volumes of liquids produce higher concentrations of long-lived reactive species (i.e., H_2_O_2_, NO_2_^−^, NO_3_^−^) [51].

For chemotherapeutic drugs, it is recognized that in vitro 2D cultures can lead to results that deviate considerably from the in vivo response [54], so as reflected in the papers reported, higher concentrations of RONS in PCL should be produced for the in vivo setting. This has been shown in Tornin et al. [55], reporting that higher concentrations of RONS are needed in 3D engineered tumor cultures in comparison to 2D cultures, and similarly in organotypical experiments with tumor sections [15]. In this sense, we strongly suggest that to relate in vitro and in vivo results, the RONS should be quantified and compared in both situations.

### 3.3. Nature of the Treated Liquid

**Figure 2:**
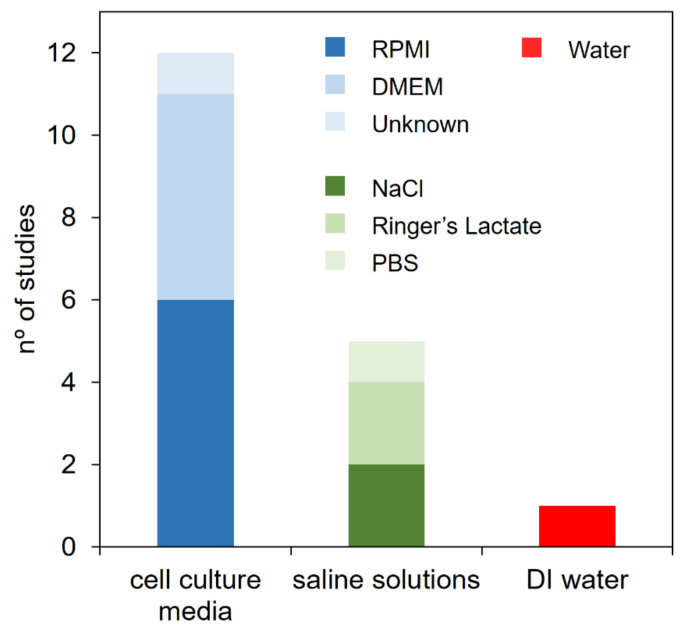
Three different kinds of liquids were used to generate PCL for in vivo assays.

Another relevant factor to take into account for the clinical translation of PCL-based therapies is the nature of the liquid employed to generate PCL. While different varieties of PCL have been investigated in vitro [51], for in vivo studies, this is more restricted. In fact, only three different liquids have been employed to date in order to produce PCL for cancer treatment in vivo: (i) cell culture media, (ii) saline solutions, and (iii) deionized water (Figure 2). The nature of the liquid has a direct effect on the amount of RONS generated after plasma treatment, which, in turn, has a clear influence over the therapeutic effect of the PCL. However, in some studies, the liquid used in vivo was different from the liquid used in vitro [19,33], and, in certain cases, the PCL employed in vivo is not clearly specified [25,30]. This means that the kind and concentration of RONS is unknown. This is a critical issue, as to give just an example in [56], PBS and 0.9% NaCl treated under equal CAP conditions displayed different anticancer activity in vitro on spheroids models of colorectal and ovarian cancer. More details about the importance of the composition of PCL with regard the generation of RONS can be retrieved in [1,2,51].

From another perspective, cell culture media (DMEM and RPMI) are surprisingly the most widely used liquids to generate PCL in the in vivo studies to date, followed by saline solutions (PBS, NaCl 0.9% and Ringer’s Lactate) (Figure 2). Cell culture media are a source of nutrients for cells to support their growth in vitro, but they are not clinically approved liquids. Therefore, for in vivo applications, saline solutions are a more interesting approach to generating PCL in view of translation to the clinics, as some saline solutions are already clinically accepted and in use in medical applications (i.e., NaCl 0.9% or Ringer’s lactate). Additionally, it has been reported that the stability of RONS is lower in plasma-treated cell culture media than in other liquids, mainly due to the reaction between the reactive species and the components in the culture media (amino acids, vitamins, proteins, glucose, etc.) that may scavenge RONS [57].

Some remaining challenges of PCL are that from a pharmaceutical point of view, the development of a liquid with anticancer properties should be able to be stored for a relatively long period of time without losing its anticancer capacity. However, in the current state of research, storage of PCL is not yet possible in most cases, so devices will have to be designed allowing the treatment and placing in the dispenser of PCL in the same surgical scenario with minimal manipulation from the surgeon/oncologist.

## 4. In Vivo Cancer Treatment with PCL

As mentioned in previous sections, it is crucial to standardize protocols to validate and increase the relevance of research works focused on the anticancer activity of PCL in in vivo approaches. In this sense, establishing consensus with regard to tumor monitoring, analytical techniques, and PCL dosage regimes should be used to make comparable the experimental data with PCL. This would allow the scientific community to advance at faster path towards clinical translation.

### 4.1. Tumor Formation

As mentioned, only grafted tumors have been used to assess the anticancer potential of PCLs in vivo. When working with grafted tumoral models, it is important to define the best engraftment strategy to represent different cancer types. In general, there are two main strategies used in tumor formation: ectopic and orthotopic. In ectopic approaches, tumors are generated in a non-native location in the animal body. Otherwise, in orthotopic models, cancer cells are injected in the native site of specific cancer where tumors are finally formed [58,59]. Comparing all PCL-based anticancer studies to date, we observe that only ectopic models have been used, mainly with subcutaneous and peritoneal tumors. Subcutaneous tumor models have been used to evaluate PCL treatments over the following cancers: melanoma [16,17,18,19,20], neck [25], breast [21,22] ovarian [23,24], and pancreatic cancers [26]. On the other hand, tumors formed in the peritoneal cavity have been used to study cancers such as pancreatic [27,28,29], ovarian [30], colorectal [33], gastric [31,32], and endometrial cancer [32].

In the majority of investigations using subcutaneous ectopic models, tumors were easily observable under the skin, and their growth was monitored with daily measurements with metric calipers. Most studies (64%) initiated administration of PCL when tumors reached a specific size range between 4.5–6 mm in diameter. This is a step forward towards establishing common protocols in the field. Unfortunately, in almost 71% of the works involving peritoneal tumors, there is no formation of solid tumors in the animal, and PCL is administered right after injection of cancer cells. Moreover, although the rest of the studies wait reasonable times to allow tumor formation, the number and size of such tumors are not quantified at the beginning of the study. This was associated to disparities among protocols such as variations between lag times to generate tumors and starting points of PCL administrations, as well as differences in the tumoral burden with the presence or not of solid tumors.

For instance, Liedtke et al. evaluated the effect of PCL on solid peritoneal pancreatic tumors that were grown for seven days before initiating administration of PCL [29]. In contrast, Sato et al. started PCL therapy just after injection of cancer cells without the presence of any solid tumor [28]. Thus, important differences exist among works, so the establishment of common procedures would certainly allow extracting more valuable conclusions. Some studies evaluating the effect of PCL on pancreatic, ovarian, and gastric cancers [27,28,30,31,32] did not allow time for the formation of solid tumors. In this context, comparing the number of injected cancer cells varies from 8 × 10^5^ to 2 × 10^6^ total cells, thus also conditioning the initial cancer cells burden, and certainly different cells will present a variable growth and tumor-forming ability. Therefore, these are some points that should be taken into account, in views of extracting more valuable conclusions: (i) having solid tumors, so allowing sufficient time after injection of cancer cells, and (ii) size of the tumor, to establish similar tumoral burden in the animal models, thereby enabling comparison among studies evaluating the therapeutic effect of PCLs.

### 4.2. Tumor Monitoring

**Figure 3:**
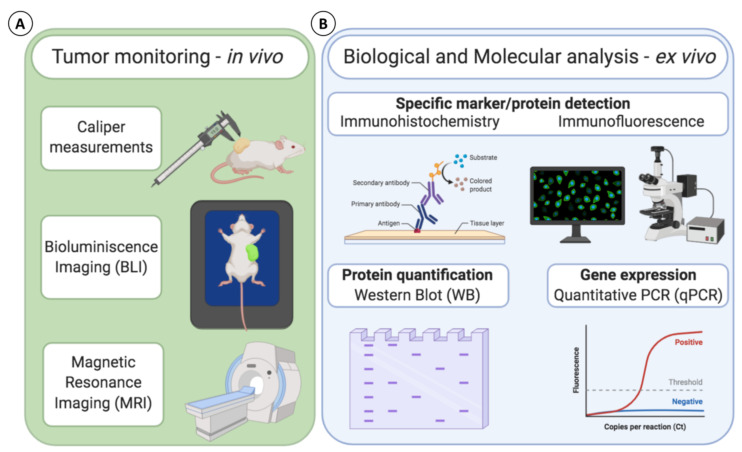
Possible methods available for tumor monitoring and biological molecular analysis to monitor and evaluate the effectiveness of PCL treatments in vivo. (**A**) Analysis applicable for tumor monitoring in vivo; (**B**) Biological and molecular analysis for ex vivo tumor analysis

As previously mentioned, establishing the initial tumoral burden in the animals is necessary to evaluate the anticancer activity of PCLs. In this sense, the number and size of tumors before initialing PCL administrations, during and at the end of the treatment, should be quantified to have precise control over the whole study. As to what concerns subcutaneous tumors, in most of the studies to date, the initial tumoral burden in the animals has been similar, and the evolution of the tumor size during the PCL treatment has also been monitored. In this case, the easy observation of tumors under the skin allows simple and painless measurements with metric calipers (Figure 3A).

However, when working with peritoneal tumors, the monitoring of tumoral burden is more difficult, so none of the investigations in the field to date have quantified the number and size of new-formed tumors at the beginning of the study. In this regard, imaging techniques that enable the quantification of tumor burden in situ—such as bioluminescence imaging (BLI) or magnetic resonance imaging (MRI)—would allow this follow-up from the first moments and provide a more complete vision of the effects of the therapy on the tumors [60]. For example, light-based methods are currently being used to visualize cell line-based tumors that have been genetically engineered to express fluorescent proteins [61] (Figure 3A). In fact, some of the reviewed studies successfully monitored the tumor burden through BLI of gastric GCIY-EGFP cancer cells expressing green fluorescence protein (GFP) [31], and pancreatic AsPC1-CMV/Luc cancer cells and ovarian ES2 cancer cells both engineered to synthesize luciferase [28,30]. However, insufficient lag time between inoculation of cancer cells and PCL treatment did not allow the establishment of the initial number and size of tumors before PCL therapy.

When wishing to increase the predictive potential and to better represent the tumor heterogeneity between patients by using PDX models, luminescent methods cannot be applied, since genetic modification may change the biological characteristics of the native tumor. Instead, MRI provides higher detection accuracy, and it does not require genetic engineering to visualize and quantify tumor burden in animals [60,62] (Figure 3A). Therefore, MRI is compatible with both cell line and PDX cancer models and would allow the precise visualization and measurement of the tumors in situ before starting PCL administrations. Moreover, MRI would be suitable also in orthotopic models, which mimic better cancer diseases by generating the tumors at the precise location of specific cancer.

### 4.3. Biological and Molecular Evaluation: Analytical Techniques

Besides the evolution of the tumor size and location, to understand the molecular basis behind the therapeutic effects of PCL in cancer treatment in vivo, it is necessary to associate biological and molecular studies. Moreover, deeper knowledge about the effect and implications of PCL on tumoral and healthy tissues will allow to evaluate and determine the safety of this novel approach in cancer treatment.

In this context, 41% of the studies evaluating PCL as anticancer agent in vivo have performed some ex vivo biological and/or molecular evaluation at the end of the study. Most of such ex vivo evaluation was performed in subcutaneous cancer models, probably due to the easier retrieval of the tumor mass compared to peritoneal models. Among them, the most used analytical technique to date has been immunohistochemistry (IHC) [17,18,22,25,27], followed by immunofluorescence (IF) [16,19,23,27], western blotting (WB) [16,17,19,25], and finally qPCR [17], which is the less used method (Figure 3B). All the mentioned techniques have been mainly used to assess the effect of PCL on cell proliferation, cell cycle arrest, and apoptosis through detection, visualization, or quantification of specific proteins and genes involved in these cellular mechanisms. Interestingly, four common proteins/genes have been evaluated through IHC, WB, and qPCR in these studies. Specifically, p53, which is involved in cell cycle arrest and apoptosis [63], Bcl-2 and Casp-8, which are associated with apoptotic pathways [64], and Akt, which is involved in cell proliferation and growth [65].

Then, more specific markers for each particular kind of tumor should be analyzed, as in Zhou et al., where the effect of PCL was evaluated on the specific markers ER, HER2, and ALDH1, which are key proteins in the development of breast cancer [22]. Even beyond, studying the specific cancer transcriptome profile would allow the scientific community to identify the gene networks regulated by PCL treatment, and correlate it to cancer gene ontology to provide a deep understanding of genomic and proteomic data of cancer and healthy cells.

Additionally, the TUNEL assay has been employed to detect fragments of DNA generated during apoptosis. In fact, Liedtke et al. used this method to evaluate the selective cytotoxic effect of PCL, where apoptosis was visualized in tumoral cells, while healthy cells appeared normal without signs of apoptosis [27].

As discussed, performing biological and molecular analysis will enable the scientific community to correlate the characteristics of PCL with their selective anticancer potential at the cellular level, which is of crucial importance to avoid incidental damage on patients and ensure the safety of this novel therapy.

### 4.4. PCL Dosage Regimen

One additional relevant point to assess in vivo efficacy of PCL lays in the dosage regimens established. PCL-based anticancer treatments have been demonstrated to be effective in vivo in murine models against pancreatic, melanoma, ovarian, breast, neck, colon, and gastric cancers by achieving a significant tumor mass reduction in most subcutaneous and peritoneal tumors. As reflected in Table 2, there are notorious differences among treatment durations, volumes of injected PCL, and their dosage regimen. For instance, disparate times of treatment ranging from three days to five weeks, different volume doses ranging between 200 and 2500 µL, and variety among the number of doses from three to seven PCL administrations per week (Table 2). In general, the biggest volumes were employed in peritoneal tumors, as the treatment area is wider, and the most commonly injected dose was 200 µL.

While the research is still in an early stage to draw general conclusions regarding the most effective PCL treatment, valuable information can be extracted. For instance, research in melanoma gathers one third of all the existing studies evaluating the anticancer activity of PCLs. In four out of the five existing studies to date, authors injected 1 × 10^6^ melanoma cancer cells and allowed tumors to reach similar sizes around 4.5–6 mm in diameter. In most studies, 200 μL of the PCL was administered daily, but with significant differences on the length of treatment that was set at three, four, 14, or 25 days. For instance, Adhikari et al. carried out the experiments with the shortest dosage regimens (three and four days). In both studies, authors performed a 10 min µ-DBD treatment to generate PCL, 200 µL of which were injected daily in the tumor site for three or four days. Three-day treatment was not sufficient to observe any tumor reduction, while in four-day PCL treatment, tumors experienced a growth reduction of around 20–30% without apparent side effects [17]. In this line, Liu et al. achieved a higher tumor growth reduction of 78.7% in a 14 days PCL treatment [19]. The longer treatment is probably not the only parameter that enhanced the anticancer effect of this treatment. Here, the authors used a plasma jet device with helium instead of a µ-DBD with air, and instead of cell culture media as vehicle for the RONS, they used 5-min-treated saline solution. In this sense, the large variability in the PCL therapies reviewed, along with the disparity in their therapeutic effects, reveals the importance of optimizing and establishing standard protocols to better understand the therapeutic effects of this novel anticancer approach.

Another issue which is of undoubted interest is the potential synergy of PCL with other entities such as drugs. In this sense, two works evaluated chemotherapeutics in combination with PCL [18]. One of them involved PCL and silymarin, a bioactive molecule extracted from milk thistle, which has demonstrated chemosensitizing activity against various cancers in vivo, as well as in clinical trials [66,67]. Results demonstrated an additional reduction in tumor growth of 50% in animals that were treated with silymarin (1 mg of/kg of body weight) one day before starting PCL treatment as compared to no tumor reduction of the PCL treatment alone. Similarly, Saadati et al. also assessed the anticancer potential of a combinational treatment involving PCL and cyclophosphamide (chemotherapeutic drug) on melanoma cancer in vivo [16]. In this case, treatment with PCL alone showed a tumor growth reduction of 76%, while the combination therapy demonstrated higher anticancer activity with a reduction of tumor growth around 86%. Importantly, the combined treatment not only arrested tumor growth, but reduced tumor volume from approximately 100 to 7 mm^3^. Although few side effects were reported in the animals, combinational therapies demonstrated a synergistic anticancer activity. Therefore, this can clearly be an interesting approach for further development.

**Figure 4:**
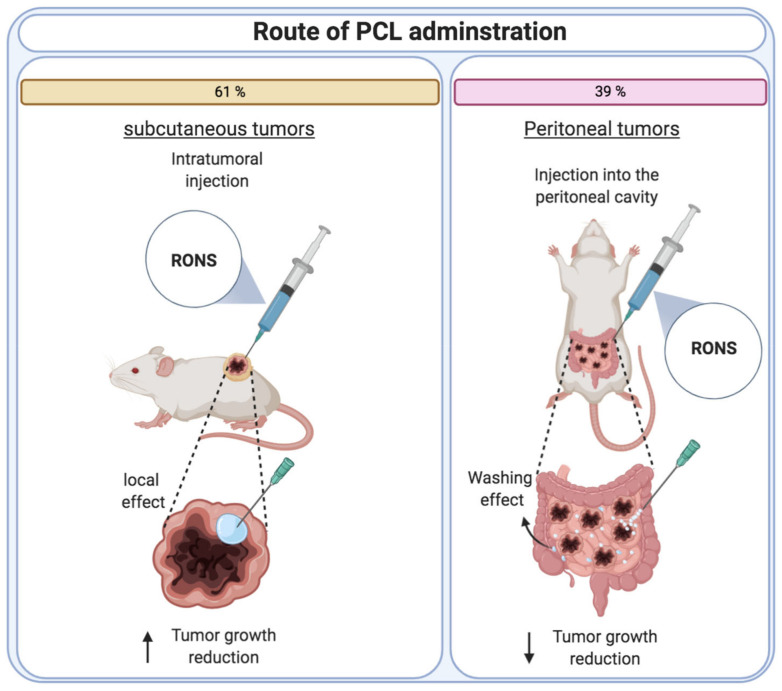
Schematic illustration of subcutaneous and peritoneal administration of PCL, including the percentage of the studies that used each route of administration to treat tumors in vivo with PCL.

The administration of PCLs in subcutaneous tumors is relatively easy, since in most cases, there is a single tumor mass that is visible under the skin, which allows an accurate and effective injection of PCL nearby the tumor mass or even intratumorally. However, in more internal and spread cancers represented by intraperitoneal tumors, multiple tumoral nodules are spread throughout the intraperitoneal cavity. Therefore, the internal tumors are not visible, and it is more difficult to equally reach them with PCLs compared to subcutaneous tumors treatment (Figure 4).

For this reason, in peritoneal tumors, the volume of PCL administered is higher than that injected in subcutaneous tumors. For instance, in two studies, authors administered 1 mL plasma-treated DMEM medium intraperitoneally to treat pancreatic cancer [27,31]. Another example can be found in the study conducted by Sato et al., where authors administered 2.5 mL of plasma-treated RPMI medium or 2.5 mL of plasma-treated Ringer’s lactate solution into the animals also suffering pancreatic cancer [28]. Despite the increase of PCLs dosage volume, in general, the anticancer activity was lower compared to the great therapeutic effect observed in the subcutaneous tumor treatments. This was confirmed in [42], where tumor burden in the animals was quantified by MRI. Here, after daily intraperitoneal administration of 1 mL of plasma-treated DMEM for 21 days, the treatment only achieved a tumor growth reduction of around 21% [27]. However, it should also be mentioned that in most studies working with intraperitoneal tumors, authors performed intermittent PCL dosing regimens with different treatment durations ranged from three days to three weeks. In this sense, due to the obvious diversity of methodologies, it is difficult to analyze the data and detect which parameters are key factors in PCL-based anticancer therapies. Therefore, although the majority of studies showed effectiveness in reducing and delaying tumor growth, there are still many open questions that require more investigation before this novel anticancer approach can be translated towards clinical application.

## 5. Conclusions and Future Directions

The field of PCL-based anticancer therapy is moving forward, as research has already shown very encouraging results in vivo in murine models. Since the concentration of RONS and dosage regimen are the two most critical assets in the efficiency of the therapy, an accurate quantification of the RONS in the conditions of the in vivo experiment is crucial. As the field progresses, standardized methods are required as much as possible, ensuring, for example, a minimum lag-time between generation of the tumor and initiation of the PCL therapy, and follow-up of the tumor size with imaging techniques during the experiment along with control of the animal’s body weight. Ultimately, a detailed ex vivo analysis of relevant biomarkers should contribute to improve the understanding related to the mechanisms of PCL, triggering their selective anti-cancer effect. Of course, the full potential of any tumor model can only be met by proposing an appropriate animal model according to the biological response to be evaluated. Therefore, the establishment of PCL’s therapeutic efficacy in preclinical testing requires a hierarchical approach, progressing through a series of animal models of increasing sophistication to eventually evidence both the efficacy and safety of PCL treatments.

PCL hold great prospects as anticancer therapy, and we need to take advantage of the lessons learnt and discussed in this review to quickly progress towards a more detailed knowledge of the mechanisms involved in the anticancer action of PCL, as well as the suitable dosage regimens and concentrations of RONS, employing liquids already in use in the clinical scenario. This will allow moving forward to the clinics in a sooner stage.

## Figures and Tables

**Table 1:** Cold atmospheric plasma settings employed for the generation of PCL for in vivo cancer treatment. Comparison between the concentration of RONS employed in in vivo studies and in their associated in vitro experiments.

Tumor type		Reference	Plasma Device/Gas	Treatment Conditions of Liquids for In Vivo Cancer Therapy					Quantification of RONS in PCL		Comments
				Gas Flow (slm)	Liquid Type	Liquid Volume (mL)	Distance (mm)	Treatment Time (min)	In Vivo	In Vitro *	
									[RONS]	[RONS] (µM)	
**Subcutaneous tumors**	Melanoma	[16]	Plasma jet/Helium	4	DMEM	1	15	6	-	-	-
		[17]	micro DBD	1.5	RPMI	-	2	10	-	-	-
		[18]	micro DBD	1.5	RPMI	-	2	10	-	-	-
		[19]	Plasma jet/Helium	3	NaCl 8.5%	1	30	1, 2, 3, 4 and 5	-	H_2_O_2_: ~ 35	Longer treatment time and different liquid type were used for the in vivo condition
		[20]	Microwave plasma generator	-	Water:DMEM (1:10)	1000	-	50	-	-	-
	Breast	[21]	Plasma jet/Helium	1	DMEM	2	13	15	-	H_2_O_2_: ~ 200	Longer treatment time was used for the in vivo condition
		[22]	Plasma jet/Helium	0.2	PBS	2	13	10	-	-	-
	Ovarian	[23]	Plasma jet/Argon	2	RPMI	4	15	10	-	-	Smaller volume of liquid and longer treatment time were used for the in vivo condition
		[24]	Plasma jet/Argon	2	Ringer’s lactate	5.5	3	10	-	H_2_O_2_: ~ 8	Smaller volume of liquid and longer treatment time were used for the in vivo condition
	Neck	[25]	Plasma jet/Helium + Oxygen	2	Serum-free culture media. Not specified	15	10–20	15	O_3_: 1.154 ppm H_2_O_2_: 1.833 ppm O_2_: 4.767 ppm NO_3_^−^: 0.167 ppm	same as in vivo	-
	Pancreas	[26]	Plasma jet/Argon	2	RPMI	4	15	10	-	-	Smaller volume of liquid and longer treatment time were used for the in vivo condition
**Peritoneal tumors**	Pancreas	[27]	kINPen Med/Argon	3	DMEM	4	5	10	-	-	-
		[28]	Plasma jet/Argon	2	Ringer’s lactate	8	3	10	-	-	Bigger volume of liquid, longer treatment time, and shorter distance of the treatment were used for the in vivo condition
		[29]	kINPen Med/Argon	5	DMEM	5	-	10	-	-	
	Ovarian	[30]	Plasma jet/Argon	2	RPMI	5.5	3	10	-	H_2_O_2_ at different dilution ratios: 0.68 to 1692.16	Not specified which dilution ratio was used for the in vivo
	Gastric	[31]	Plasma jet/Argon	2	RPMI	6	3	5	-	-	-
		[32]	Plasma jet/Argon	2	DMEM	8	3	5	H_2_O_2_: 227 µM	same as in vivo	-
	Colon	[33]	kINPen/Argon	5	NaCl 0.9%	50	-	60	-	H_2_O_2_: 100 O_2_^−^: (Abs. 0.08) NO_2_^−^: 2.5 NO_3_^−^: 8	Different liquid type was used for the in vivo condition
	Endometrial	[32]	Plasma jet/Argon	2	DMEM	8	3	5	H_2_O_2_: 227 µM	same as in vivo	-

* Note that the RONS quantified in vitro might have been produced under different conditions from these shown in Table 1, which correspond to those used in in vivo experiments.

**Table 2:** Therapeutic effects of PCL in vivo. Influence of initial tumor size, dosage regimen, and treatment duration on tumor growth reduction for different types of cancer.

Tumor type		Reference	Initial Tumor Size (mm Ø)	PCL	Dose Volume (μL)	Administration Route	Dosage Regimen	Treatment Duration (Days)	Therapeutic Effect	
									Tumor Growth Reduction (%)	
**Subcutaneous tumors**	Melanoma	[16]	5–6	DMEM	400	Subcutaneous	Daily	25	76.7 *	
		[17]	4.65	RPMI	200	Subcutaneous	Daily	4	≈20	
		[18]	4.65	RPMI	200	Subcutaneous	Daily	3	No reduction **	
		[19]	5	NaCl 8.5%	200	subcutaneous (intratumoral)	Daily	14	78.7	
		[20]	Not quantified	Water:DMEM (1:10)	1000	Subcutaneous	Daily	12	≈85	
	Breast	[21]	5	DMEM	200	Subcutaneous	Daily	30	≈80	≈10 ^a^
		[22]	5	PBS	200	Subcutaneous	Daily	30	≈60	
	Ovarian	[23]	No tumor formation	RPMI	200	Subcutaneous	3 times/week	30	≈66.7	≈37.5 ^a^
		[24]	No tumor formation	Ringer’s lactate	200	Subcutaneous	3 times/week	42	≈75	
	Neck	[25]	4.65	Serum-free culture media. Not specified	200	Subcutaneous (intratumoral)	Daily	6	≈50	
	Pancreas	[26]	No tumor formation	RPMI	200	Subcutaneous	3 times/week	30	≈66.7	
**Peritoneal tumors**	Pancreas	[27]	No tumor formation	DMEM	1000	Intraperitoneal	Daily	35	≈21.1	
		[28]	No tumor formation	Ringer’s lactate	2500	Intraperitoneal	Days 2–4 and Days 8–11	15	Not quantified	
		[29]	Not quantified	DMEM	1000	Intraperitoneal	Daily	21	≈27.8	
	Ovarian	[30]	No tumor formation	RPMI	Not specified	Intraperitoneal	Daily	3	Not quantified	
	Gastric	[31]	No tumor formation	RPMI	Not specified	Intraperitoneal	Days 1–4 and Days 8–11	15	Not quantified	
		[32]	No tumor formation	DMEM	1000	Intraperitoneal	Days 0, 1, 2, 6, 7, and 8	8	Not quantified	
	Colon	[33]	Not quantified	NaCl 0.9%	300	Intraperitoneal	Days 2–6	Not specified	≈66.7	
	Endometrial	[32]	No tumor formation	DMEM	1000	Intraperitoneal	Days 0, 1, 2, 6, 7, and 8	8	Not quantified	

* Co-treatment with cyclophosphamide 130 mg/kg intraperitoneal led to a tumor size reduction of 98%. ** Co-treatment with silymarin 1 mg/kg led to a tumor growth reduction of 50%. ^a^ Two different tumor growth reductions are displayed, because two different cell lines were used for these experiments.

## Data Availability

Not applicable.
